# Advancements in Tumor-Targeted Nanoparticles: Design Strategies and Multifunctional Therapeutic Approaches

**DOI:** 10.3390/nano15161262

**Published:** 2025-08-15

**Authors:** Mengya Li, Shengxi Zhou, Yan Zhang, Jingan Li, Kun Zhang

**Affiliations:** 1 School of Life Science, Zhengzhou University, Zhengzhou 450001, China; limengya@gs.zzu.edu.cn (M.L.); zhoushengxi@gs.zzu.edu.cn (S.Z.); yanhuo@gs.zzu.edu.cn (Y.Z.); 2 School of Materials Science and Engineering, Zhengzhou University, Zhengzhou 450001, China

**Keywords:** nanoparticles, surface modification, synergistic therapeutic effects, diagnosis

## Abstract

Cancer treatment faces significant challenges due to drug resistance, non-specific toxicity, and limited penetration of therapeutic agents. Here, we discuss the latest advancements in the design and application of tumor-targeted nanoparticles, focusing on polymer-based, biomimetic, and inorganic nanocarriers, as well as innovative surface modification strategies, to enhance diagnostic and therapeutic approaches in cancer treatment, including the co-delivery of chemotherapeutic agents with biologicals or photo/sonosensitizers for synergistic therapeutic effects. This review not only highlights the current importance of nanoparticle design and application for tumor targeting but also provides insights into future directions for more effective cancer therapies. By integrating advanced material science with biology, these strategies hold the potential to transform the landscape of cancer treatment, offering hope for improved patient outcomes and personalized therapeutic approaches.

## 1. Introduction

Nanomedicine is an emerging multidisciplinary field that is attracting scientists [1,2]. It holds great potential in cancer treatment and tracing tumor cells [3]. This involves designing suitable nanocarrier constructs to deliver precise therapeutics—including chemicals [4] and gene segments [5]—to target tumor cells via both active and passive mechanisms, with the aim of enhancing therapeutic efficacy while simultaneously minimizing damage to healthy cells [6]. On the other hand, it can act as the fastener, which loads photosensitizers [7,8] and sonosensitizers [9], to achieve radiosensitization and work in conjunction with photothermal therapy [10]. Nanoparticles also function as tracer agents in diagnostics. When conjugated with fluorescent groups, they enable high-sensitivity, high-resolution optical imaging. Alternatively, their magnetic properties facilitate imaging in magnetic resonance imaging (MRI) [11,12]. New approaches to tumor labeling and detection are also possible using nanoprobes and nano-biosensors [13].

**Figure 1:**
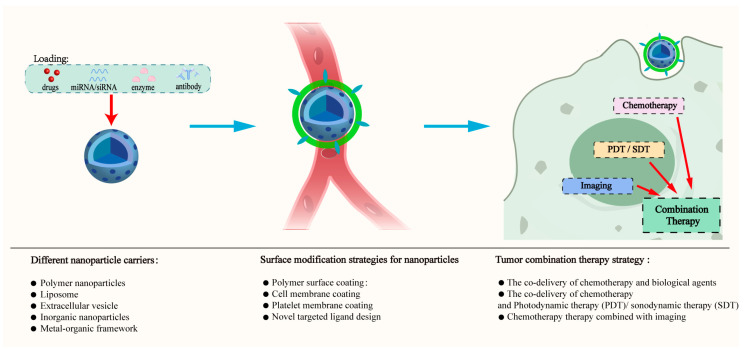
Flowchart of design strategy for multifunctional nanoparticles.

Selecting engineered bio-nanoparticle carriers, coupled with different surface modification strategies to increase their functions, enables the targeting of tumors in exquisite cascade responses or combination therapies, as shown in Figure 1. This review critically analyzes advanced design strategies for multifunctional nanocarriers, focusing on a comparative evaluation of co-delivery systems across polymeric, lipidic, biomimetic, inorganic, and MOF-based platforms while dissecting innovative surface engineering approaches and multimodal therapeutic strategies that enable spatiotemporal control. It also provides a brief discussion of the prospects and challenges.

## 2. Methodology

This review synthesizes research identified through comprehensive searches of ScienceDirect, Web of Science, and PubMed using Boolean operators with keywords including “polymeric,” “extracellular vesicle,” “drug delivery,” “nanomaterials,” and “liposome”. The analysis focuses on nanomaterial applications in tumor therapy, encompassing classification and functional characteristics, nanoparticle toxicity profiles, and clinical translation.

**Figure 2:**
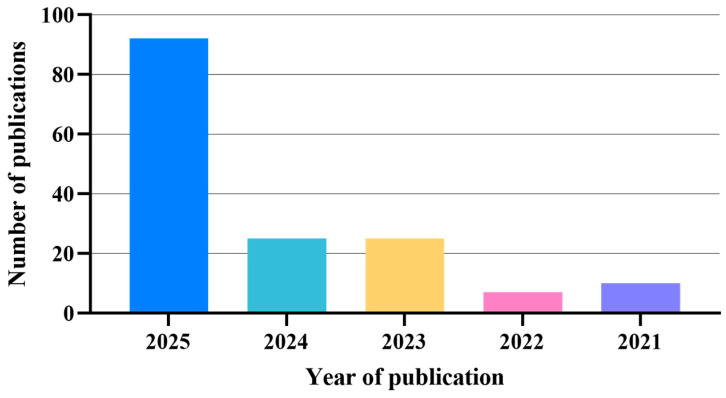
The correlation between the number of publications and year of publication of the research covered in this review.

Publications spanning 1982 to the present were evaluated, with heightened emphasis on recent advances from 2021 to 2025 (134 of 199 references). Figure 2 illustrates the temporal correlation between annual publication volume and research progression during this five-year period. This review systematically examines nanocarrier selection, surface modification strategies, and functional design principles for combination therapies in tumor treatment.

## 3. Different Nanoparticle Carriers

Nanocarriers form the foundation of bioengineered nanoparticles. Ideal bioengineered nanoparticles should satisfy some fundamental criteria, including a composition or structure enabling rapid response to the stimulus, a stable size and low polymer dispersity index (PDI), high carrying capacity, excellent biocompatibility, and controlled degradability [14]. Some typical nanocarriers are listed as follows: polymer-based nanocarriers, including natural polymer nanocarriers and synthetic polymer nanocarriers; biomimetic-based nanocarriers, including liposomes and protein nanoparticles; inorganic nanocarriers, including mesoporous iron nanoparticles; and other nanocarriers including metal–organic frameworks (MOFs).

### 3.1. Polymer Nanoparticles

Polymer nanoparticles typically range from 10 to 200 nm in diameter, though larger-sized ones are occasionally reported [15]. Conventionally, they are classified as either synthetic or natural polymers; these nanoparticles possess distinct characteristics that enhance their performance in drug delivery systems [16].

#### 3.1.1. Natural Polymer Nanoparticles

Chitosan is a kind of natural polymer nanoparticle which is water-soluble, biocompatible, and biodegradable [17] and is derived from the shells of crustaceans [18,19]. It is the most utilized natural polymer nanoparticle in nanomedicine to date. Due to its unique transmucosal properties, chitosan-based biomaterials are extensively employed in drug delivery systems targeting various epithelial tissues (e.g., the eyes [20] and the gut [21]). This enables oral administration, widely regarded as the most patient-compliant and convenient delivery route. Chitosan facilitates drug penetration through the mucosal epithelium by reversibly opening tight junctions between epithelial cells. This paracellular transport allows therapeutic cargo to cross mucosal barriers and reach target tissues like tumors [14]. Moreover, derivatives developed using chitosan are able to compensate for inherent bad solubility deficiencies while retaining permeability [22]. Chitosan has been reported to activate NLRP3 inflammatory vesicles, leading to a robust IL-1β response through a phagocytosis-dependent mechanism [23]. Flávia Castro et al. reported a Ch/γ-PG nanoparticle that enhances T cells and kills tumor cells by stimulating low immune-responsive macrophages and immature dendritic cells to move to the high immune response stage [24]. However, significant limitations challenge clinical translation. Although chitosan nanoparticles accumulate in tumors via the EPR effect, tumor targeting efficiency remains low and non-specific accumulation in normal organs is high. In vitro, 900 μg/mL Cy5.5- chitosan nanoparticles induce H9C2 cardiomyocyte necrosis and pro-inflammatory cytokine release (TNF-α, IL-1β, IL-6) [25]. In vivo, intravenous administration (90 mg/kg) in mice shows persistent blood retention (>72 h) and cardiac accumulation, causing dose-dependent cardiotoxicity with histopathological damage and organ dysfunction following repeated dosing. Embryotoxicity studies in Danio rerio reveal that chitosan nanoparticles cause dose-dependent developmental abnormalities, including 24–120h post-fertilization hatching delays and teratogenic effects [26]. Notably, this dose-dependent toxicity arises primarily from non-targeted accumulation of chitosan nanoparticles in multiple organs, consistent with findings by Wang et al. Importantly, at concentrations ≤ 250 mg/L (equivalent to mammalian intravenous doses of ~22.5 mg/kg), chitosan nanoparticles exhibit significantly lower embryotoxicity [25]. Specifically, chitosan nanoparticles at 250 mg/L induce only 25.0% mortality at 120 h post fertilization (hpf) versus 44.4% for conventional chitosan particles, alongside markedly reduced teratogenicity. Furthermore, chitosan nanoparticles at ≤150 mg/L limit mortality to 8.3% and malformation to <10%, with 72 hpf hatching rates comparable to controls. This cross-species dose consistency underscores conserved biological responses to nanoparticles [25,26]. Consequently, rigorous preclinical validation of dosing regimens and patient stratification—particularly contraindication in pregnant women and women of childbearing potential—is essential before clinical deployment to mitigate these adverse effects.

Additionally, nanotoxicity is governed not only by administered dose but also by physicochemical properties (e.g., chemical composition, size, shape). For instance, Bai et al. demonstrated that nanoparticle core hydrophilicity/hydrophobicity critically influences cellular uptake [27], while Bar-Ilan et al. established material-dependent toxicity (gold nanoparticles < silver nanoparticles) [28]. Talamini et al. further elucidated how size and shape dictate biodistribution, accumulation, and clearance in murine models [29]. However, these findings remain material-specific, necessitating carrier-specific assessments of size, shape, and composition to predict off-target organ accumulation and intracellular toxicity.

Another widely explored natural polymer-based nanocarrier is protein nanoparticles, exemplified by human serum albumin (HSA). HSA offers several key advantages for drug delivery [14]. (1) Endogenous Origin: As a natural carrier within the organism [30], HSA minimizes risks of inflammation and associated toxicity. (2) Natural Transport Function: Its prolonged systemic circulation enables sustained drug release kinetics [30]. (3) Extended Circulation: Its prolonged systemic circulation enables sustained drug release kinetics. (4) Biocompatible Metabolism: HSA is biodegradable, yielding harmless metabolites suitable for reuse [31]. (5) Tumor Targeting: It exhibits strong enhanced EPR effects in solid tumors, making it an ideal therapeutic vehicle [30]. (6) Active Tumor Uptake: Cancer cells express HSA-specific receptors (e.g., SPARC, gp60), facilitating receptor-mediated endocytosis [32,33]. Novel recombinant fusion proteins from HAS domain III demonstrate high affinity for human esophageal cancer cells. These constructs achieve tumor site-specific biodistribution in vivo, with targeted enrichment persisting for up to 624 h [34]. HSA nanoparticles can be further functionalized to enhance tumor cell uptake. Xiong et al. enhanced tumor cell targeting through lysine acetylation of HSA nanoparticles, promoting specific CD44 receptor binding and fluid-phase macropinocytosis for non-specific internalization [35]. Alternatively, conjugating immunoadjuvants to HSA nanoparticles via active amino groups enables glutathione (GSH)-responsive activation within tumor cells, and co-loading of the oncotic peptide forms the iP-RS nanoparticles. This system synergistically reconfigures the tumor microenvironment and triggers potent immune responses [36]. Clinical validation comes from FDA-approved Abraxane^®^, demonstrating HSA’s therapeutic potential. However, clinical translation faces scalability constraints: the limited availability of human serum albumin and challenges in purifying plant-derived alternatives necessitate novel extraction methodologies to streamline production.

To sum up, future advancements in natural polymer nanomedicine depend on rationally engineered designs to overcome existing translational barriers. For chitosan, optimizing surface functionalization and ligand conjugation is crucial to mitigate non-specific accumulation and organ toxicity while enhancing tumor targeting. Addressing HSA’s scalability requires innovative, sustainable production platforms. Harnessing deeper insights into nanoparticle–biointerfacial interactions and carrier-specific property–toxicity relationships will guide the development of safer, more effective next-generation systems. Despite current challenges, the intrinsic biocompatibility and functional versatility of these polymers render their clinical translation highly promising, particularly through continued innovation in structural modification and targeted delivery strategies.

#### 3.1.2. Synthetic Polymer Nanoparticles

**Figure 3:**
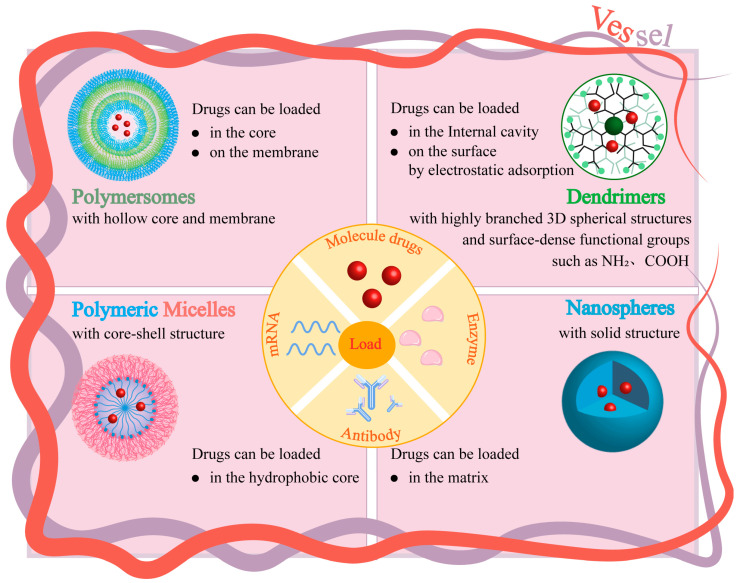
Four kinds of polymer nanoparticles.

Synthetic polymer nanoparticles are extensively utilized in tumor drug delivery, Based on their structure, mode of assembly, and material properties, they are classified into four categories: polymersomes, dendrimers, polymeric micelles, and nanospheres [37]. Drugs can be incorporated into these nanocarriers through encapsulation within the core area or the matrix and chemically coupled or bound to the surface of the nanoparticle. This versatility enables synthetic polymer nanoparticles to transport diverse therapeutic cargoes including hydrophobic/hydrophilic compounds and molecules of varying weights, ranging from chemotherapeutic agents to biomolecules like proteins, mRNAs, and antibodies (Figure 3).

Polymersomes are artificial vesicles formed through the self-assembly of synthetic amphiphilic block copolymers. While structurally analogous to liposomes—characterized by phospholipid bilayers with hydrophilic head groups and hydrophobic tails—polymersomes fundamentally differ in their synthetic composition. This architecture confers superior stability and enhanced drug-loading capacity compared to conventional liposomes, with hydrophilic therapeutics encapsulated in the aqueous core and hydrophobic agents embedded within the membrane bilayer, enabling efficient cytoplasmic delivery. FDA-approved copolymer components like polylactic acid (PLA) [38] and poly(ethylene glycol) (PEG) [39] facilitate clinical translation. Hao et al. developed near-infrared-responsive 5-Fu-ICG-MPEG-PCL polymersomes co-loaded with 5-fluorouracil and indocyanine green, integrated into microneedle systems for treating epidermoid carcinoma and melanoma [40]. Shang et al. engineered photothermal agent-loaded vesicles analogous to polymersomes, achieving high photothermal conversion efficiency with demonstrated biosafety for bladder cancer therapy [41]. Cai et al. designed poly(2-methyl-2-oxazoline) (PMOXA)-based polymersomes that outperform PEGylated nanoparticles through enhanced drug-loading capacity and improved cellular internalization via surface-adsorbed proteins (e.g., fibronectin), promoting tumor-selective accumulation [42].

Polymeric micelles, typically formed by block copolymer self-assembly into nanospheres with hydrophobic cores and hydrophilic coronas, differ structurally from polymersomes by lacking distinct bilayer membranes for hydrophobic drug encapsulation. This architecture protects hydrophobic payloads from aqueous degradation while prolonging systemic circulation. They accommodate diverse therapeutics—from small molecules [43,44] to proteins [45,46,47]—and are actively investigated in clinical oncology trials. Cellular internalization occurs primarily via endocytosis, followed by intracellular degradation [48]. Liu et al. developed self-assembled cabazitaxel-carboxymethylcellulose-mPEG micelles through acetylated carboxymethyl chitosan modification to enhance mPEG grafting rates, while innovatively quantifying drug-loading efficiency [49]. These systems demonstrate adequate hemocompatibility, enabling delivery to most tumor types, yet face critical limitations: the inability to cross biological barriers intact restricts their application in brain tumors and fibrotic malignancies. Despite promising preclinical results, few micellar formulations have reached clinical trials or market approval due to insufficient therapeutic efficacy in human trials [48]. Consequently, redesign strategies focusing on barrier penetration and tumor-specific activation are essential to advance clinical translation.

Dendrimers are precisely engineered hyperbranched polymers with well-defined three-dimensional architectures. Their mass, size, shape, and surface chemistry can be systematically controlled during synthesis. Reactive surface functional groups enable covalent conjugation of biomolecules or contrast agents [50], while internal cavities allow for drug encapsulation. Unlike physical encapsulation, therapeutic agents can be strategically localized within distinct structural compartments: the central core, inner shells, or outer shells. The multivalent surface facilitates high-density modifications, enabling simultaneous conjugation of targeting ligands, therapeutics, and diagnostic markers to create multifunctional nanoplatforms. This functionalization permits pH- or enzyme-responsive controlled drug release in physiological environments [51,52]. To reduce selenium-associated toxicity, Sanz del Olmo et al. doped selenium into dendrimer cores and branches while preserving peripheral sites for PEGylation, conferring potent anticancer activity against breast cancer cells [53]. Qin et al. designed pH-responsive nanocomplexes with aPD-1 and paclitaxel (PTX) loaded in internal cavities, while surface-modified with aPD-1 and BSA to enhance targeting for chemo-immunotherapy [54]. These two design strategies exemplify how distinct modifications to the dendrimer’s interior and surface can confer advanced functionality, highlighting their exceptional design flexibility and vast application potential. Clinically, phase II trials demonstrate promising outcomes for dendrimer-based irinotecan and cabazitaxel delivery, showing encouraging anti tumor activity and acceptable tolerability profiles. Although manageable adverse effects warrant further clinical evaluation, these results underscore dendrimers’ significant translational potential in oncology.

In summary, synthetic polymer nanoparticles demonstrate remarkable versatility in tumor drug delivery through diverse architectures. Future development should prioritize rational engineering to overcome translational barriers—particularly enhancing micellar penetration of biological barriers and advancing dendrimer-based multifunctional platforms. Optimizing barrier-penetrating capabilities and tumor-specific activation mechanisms will be crucial for developing next-generation clinically viable systems, ultimately bridging the gap between preclinical promise and therapeutic reality.

### 3.2. Liposomes

**Figure 4:**
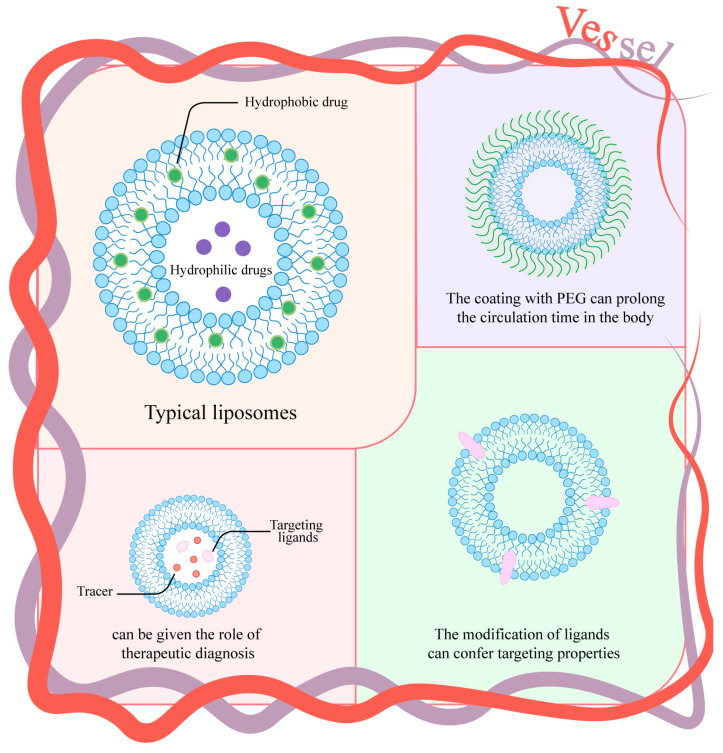
Classical structure of liposomes and several liposome types.

Liposomes are spherical vesicles (50–450 nm) comprising a phospholipid bilayer with hydrophilic head groups and hydrophobic tails surrounding an aqueous core. Structurally analogous to polymersomes, their dual-compartment architecture enables versatile drug loading: hydrophilic therapeutics encapsulate within the aqueous core while hydrophobic drugs partition into the lipid bilayer [55] (Figure 4). FDA-approved liposomal formulations play irreplaceable roles in RNA delivery and combination therapy [56]. Notably, Doxil^®^—first approved in 1995—demonstrates enhanced efficacy when combined with hyperbaric oxygen (HBO) therapy, which overcomes tumor hypoxia to increase chemosensitivity [57]. This strategy of innovatively modifying approved drugs to improve therapeutic outcomes exemplifies a promising research direction for existing products, offering valuable insights for future development. Clinical trials of nano liposomal irinotecan show favorable biodistribution with minimal adverse events [58]. Traditional liposomes consist solely of phospholipids, while PEGylated variants feature polyethylene glycol surface coatings that prolong circulation half-life [59]. Tumor targeting is achieved by conjugating ligands (e.g., peptides [60], antibodies [61], vitamins [62], glycans [63]), or encapsulating theranostic agents [64]. However, stability limitations impede large-scale applications: even at 4 °C, vesicles undergo shape deformation, osmiophility loss, and ghost formation within 1–2 months [65]. These physicochemical instabilities—coupled with sterilization challenges—necessitate formulation optimization through cryoprotectant screening, lyophilization protocols, and continuous manufacturing innovations to enable cost-effective production and expanded clinical deployment.

Above all, liposomes offer versatile drug-loading capabilities and proven clinical utility, particularly in combination therapies. Future advancements must prioritize overcoming stability limitations and sterilization challenges through optimized lyophilization protocols and continuous manufacturing innovations. Developing robust, cost-effective production methods will be essential to expand their clinical translation and maximize therapeutic potential across broader oncology applications.

### 3.3. Extracellular Vesicles

Extracellular vesicles (EVs) are cell-derived nanoparticles enclosed by a lipid bilayer membrane [66]. Distinct from liposomes, EVs carry diverse bioactive components and were initially regarded as carriers of metabolites, waste products, or cellular debris, though their functions have been progressively elucidated [67]. Exosomes, a major EV subtype, are single-membrane vesicles (30–200 nm diameter) released by cells and contain proteins, lipids, and nucleic acids [68,69]. They represent the first identified and remain among the most clinically significant EVs [68]. Owing to their natural cell origin, EVs exhibit high biocompatibility and intrinsic targeting capabilities, making them ideal for delivering fragile biological therapeutics like oligonucleotides [70,71]. EV-mediated RNA delivery was initially considered a breakthrough in nanomedicine [72].

Immunotherapy—defined as activating or enhancing an organism’s anti-tumor immune response through immunotherapeutic agents [73,74]—has attracted increasing attention over the past decade [75]. Among the various sources of extracellular vesicles, tumor-derived extracellular vesicles (TEVs) and immune cell-derived extracellular vesicles are considered promising new directions in tumor immunotherapy [76]. TEVs are characterized by the presence of multiple immunogenic molecules on their surface including Hsp 70, miRNA, and MHC-1, that can induce an immune response for cancer cells and can be developed into novel tumor vaccines [74,77].

Similarly, the biological origin of extracellular vesicles introduces several challenges: biological background interference [78], difficulties in isolation, purification and mass production [67], intrinsic batch-to-batch variations in surface composition, poorly characterized surface ligand functionality, and hepatic accumulation [14]. Emerging research on EVs continues to advance solutions to these limitations, highlighting their significant diagnostic and therapeutic potential in oncology.

In summary, extracellular vesicles offer exceptional biocompatibility and intrinsic targeting for delivering fragile therapeutics, holding transformative potential in cancer immunotherapy. Future research must overcome isolation, scalability, and functional characterization challenges through standardized production protocols and improved biodistribution control. Addressing these limitations will unlock EVs’ full capacity for developing novel immunotherapeutic paradigms, particularly in personalized tumor vaccines.

### 3.4. Inorganic Nanoparticles

**Figure 5:**
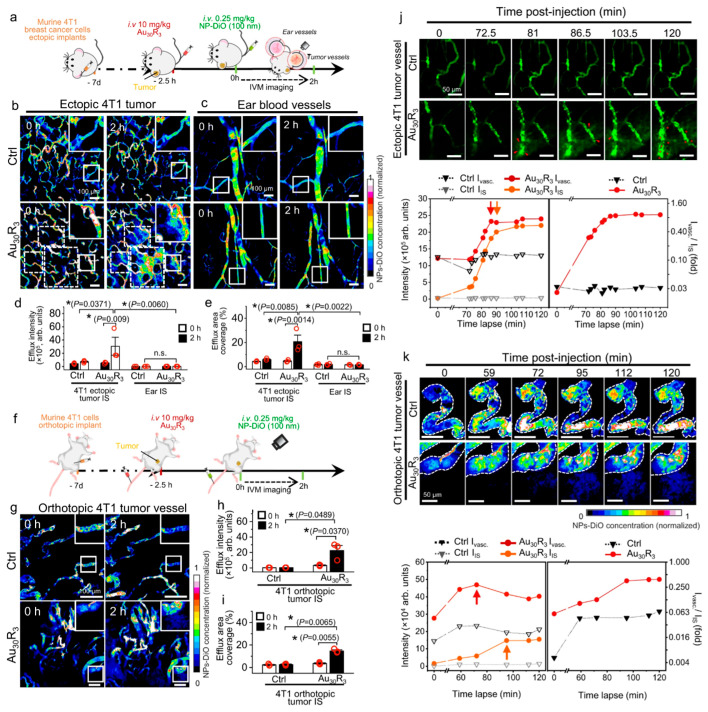
NanoEL gold nanoparticles induce tumor-specific vascular leakiness and enhance interstitial infiltration captured by real-time intravital imaging. (**a**) A schematic of NanoEL pretreatment enhancing tumoral access; (**b**) intravital imaging of NanoEL-induced leakiness in ectopic tumor; (**c**) no leakiness in healthy vasculature confirms tumor specificity; (**d**) quantified efflux intensity shows NanoEL-enhanced leakage; (**e**) increased interstitial coverage by NanoEL-induced leakage; (**f**) qhole-tumor section demonstrates deep tracer penetration; (**g**) higher tracer coverage in NanoEL-treated tumor sections; (**h**) intensified tracer accumulation in tumor core with NanoEL; (**i**) RBC extravasation confirms vascular barrier disruption; (**j**) CD31 staining reveals inter-endothelial gaps post NanoEL application; (**k**) quantified RBC infiltration into tumor interstitium. Adapted from [85], with permission from Springer Nature (2023).

Inorganic materials such as gold, iron, copper, and silica have been used to synthesize nanoparticles for drug delivery and imaging [79,80,81,82]. These inorganic nanoparticles can be engineered into a wide variety of sizes, structures, and geometries [83]. Gold nanoparticles are one of the most popular drug carriers [84]. Magdiel et al. innovatively engineered gold nanoparticles specifically designed to overcome a fundamental limitation in cancer therapy: insufficient tumoral vascular permeability. Their breakthrough lies in demonstrating that these nanoparticles, termed NanoEL, can actively and therapeutically modulate vascular integrity at the tumor site (Figure 5) [85]. Crucially, real-time intravital microscopy in live animal models (Figure 5b–e) provided direct visual evidence that NanoEL gold nanoparticles induced significant leakage specifically within tumor vasculature walls, facilitating markedly improved infiltration of co-administered fluorescent nanoparticle tracers (used as drug surrogates) into the tumor interstitial space. This engineered leakiness occurred without discernible toxicity to endothelial cells. This approach represents a paradigm shift, moving beyond solely enhancing drug cytotoxicity to strategically engineering transient vascular permeability, thereby significantly improving tumor accessibility for therapeutics. The controllable and tumor-specific nature of NanoEL-induced leakiness holds profound significance for enhancing drug delivery efficacy, particularly in tumors with poor inherent permeability (e.g., early-stage or hypovascular tumors), and potentially for targeting micrometastases.

Iron-based nanoparticles represent a strategically important class of inorganic oncology materials, primarily functioning as potent ferroptosis inducers. Fe^2+^-based nanoparticles exploit the acidic tumor microenvironment (TME), releasing active Fe^2+^ ions that catalyze the Fenton reaction. This process generates highly toxic hydroxyl radicals—a key reactive oxygen species (ROS)—which induce phospholipid peroxidation [86], and oxidize cellular macromolecules to trigger apoptosis [87]. This intrinsic TME-responsiveness is a significant advantage for tumor-specific toxicity. Xuan et al. used hepatocellular carcinoma cell membrane-encapsulated PLGA and FePt nanoparticles and loading with Lenvatinib for targeted and enhanced iron death/apoptosis, convincingly demonstrated in vitro and in vivo [88]. This biomimetic approach notably addresses passive accumulation limitations through homologous targeting. Similarly, shi et al. engineered a ferrocene (Fc) delivery system using NIR-responsive Cu-based nanoparticles coated with lauric acid and Fc [89]. This design intelligently combines stimuli responsiveness (photothermal-triggered release) with multimodal therapy (PTT/ferroptosis), showcasing an emerging trend toward complex, externally activatable platforms. Other inorganic nanoparticles, including copper and mesoporous silica, further expand this repertoire for combined therapy and monitoring [90,91,92,93], exploiting unique properties like magnetism, radioactivity, or plasmonics for diagnostics, imaging, and photothermal applications [83,94,95,96].

Over all, while inorganic nanoparticles offer superior stability, tunable multifunctionality, and theranostic capabilities often unattainable with organic materials [92], critical limitations persist. Their clinical translation is significantly hampered by long-term toxicity concerns, particularly for heavy metals (e.g., Cu, Pt, Au), which may cause off-target organ accumulation and inflammatory responses. Cost and complex synthesis of noble metal formulations present additional barriers. Future efforts must prioritize toxicity mitigation strategies—such as improved biodegradability, enhanced targeting specificity to reduce systemic exposure, and rigorous long-term biosafety studies—alongside scalable production methods to harness their full therapeutic potential.

### 3.5. Metal–Organic Frameworks

Another synthetic polymer scaffold that has been extensively studied is MOFs. Metal–organic frameworks are a class of hybridized porous materials consisting of organometallic ions combined with organic ligands [97]. Due to their porous structure and diverse functionalities, MOFs show great potential for applications in drug delivery. MOFs have a high surface area and large porosity, and therefore have good drug-carrying capacity [98]. Excellent chemical/thermal stability and the ability to respond to pH allows MOFs to release a drug under specific conditions, which can help to functionalize designs for different needs [99]. In addition, MOFs are formed of organic ligands covalently bound to varieties of metal center ions; some of the MOFs based on different ions are listed in Table 1. The release of these ions can disrupt intracellular ionic homeostasis after the structural collapse of MOFs, which can inform the design of special pathways in cells [100].

**Figure 6:**
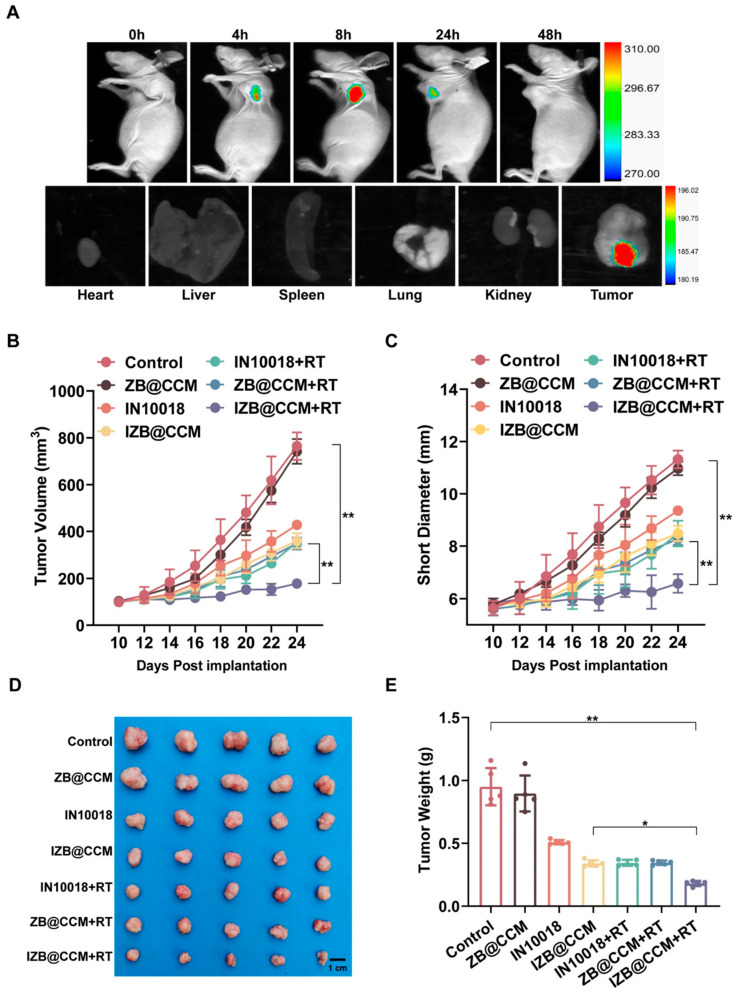
A biomimetic ZIF-8 nanoplatform for dual-targeted radiosensitization of cervical cancer. (**A**) The nanoplatform was constructed via one-pot synthesis of an IN10018/Bi-loaded ZIF-8 core and hybrid membrane coating for dual targeting; (**B**) in vivo fluorescence imaging demonstrates specific tumor accumulation of Cy7-labeled nanoparticles peaking at 8 h; (**C**) radiotherapy combination potently suppresses tumor growth, with an 82.3% volume reduction in the synergistic treatment group; (**D**) excised tumor weights verify minimal tumor burden in the synergistic treatment group, outperforming monotherapies; (**E**) IHC shows downregulated α-SMA expression, confirming CAF infiltration reduction via FAK signaling inhibition. Adapted from [103], with permission from Springer Nature (2025).

Zeolitic Imidazolate Frameworks (ZIF-8), composed of Zn^2+^ and 2-methylimidazole, are widely explored in oncology for their high surface area, pH-responsive biodegradation, and biocompatibility. These properties enable efficient co-delivery of therapeutic agents (e.g., drugs, photosensitizers) to tumors, enhancing treatment efficacy [102]. For instance, Li et al. encapsulated glucose oxidase and platinum nanozymes (GOx-Pt) in ZIF-8, leveraging its pH-sensitive degradation to trigger tumor-specific glucose depletion and ROS generation [112]. Chang et al. engineered a biomimetic dual-targeting strategy by co-loading the FAK inhibitor IN10018 and radiosensitizer bismuth (Bi) into ZIF-8 nanoparticles, followed by coating with hybrid membranes derived from cancer-associated fibroblasts (CAFs) and cancer cells. This innovative design (Figure 6A–D) achieved (i) precise dual homing to both tumor cells and CAFs via membrane protein retention, (ii) pH-triggered release of IN10018 to suppress FAK-mediated CAF infiltration and radiosresistance, and (iii) Bi-enhanced radiation absorption for synergistic radiosensitization. Real-time fluorescence imaging confirmed rapid tumor accumulation within 8 h, while in vivo studies demonstrated significant tumor regression (82.3% growth inhibition) compared to monotherapies when combined with radiotherapy [103] (Figure 6). This work exemplifies the emerging trend of “stromal cell reprogramming” using multifunctional nanocarriers to overcome microenvironmental barriers in radioresistant cancers.

To clarify the advantages and disadvantages of various nanoparticles, this paper summarizes the main strengths and limitations of the key nanocarrier systems discussed (polymeric, liposomal, MOFs, inorganic) in Table 2.

To sum up, certain MOF materials induce metal ion toxicity due to off-target biodistribution. However, effective surface modification strategies can substantially mitigate this biosafety concern. These approaches fundamentally enhance target specificity, reducing non-specific accumulation in metabolically active organs while preserving the cytotoxic potency of released metal ions—a paradoxical advantage for oncotherapy. Specifically, liberated Fe (inducing ferroptosis [86,110]), Cu (triggering cuproptosis [91]), or other catalytic ions (mediating Fenton-like reactions [115,116]) can be strategically integrated as functional components within nanoplatforms. This design enables orchestrated cascade reactions, amplifying therapeutic efficacy against malignancies.

## 4. Surface Modification Strategies for Nanoparticles

Although nanoparticles are effective carriers for drugs or other biomolecules, they need to be improved to achieve specific localization and targeted delivery, increased cellular uptake and internalization, selective recognition, non-cytotoxicity, and increased payload binding capacity [117]. To address the need for precise drug delivery and enhanced therapeutic efficacy, nanoparticles are increasingly tailored to specific therapeutic strategies [118,119]. In this focused article, we will explore some of the latest research in nanoparticle surface engineering. These studies highlight the importance of the functionalization of nanoparticle surfaces and the biomedical applications of these particle systems.

### 4.1. Polymer Surface Coating

Polymer coatings enhance nanoparticle stability by improving hydrophilicity and suspension properties, as demonstrated by PEG and chitosan modifications [120]. These coatings—particularly polyethylene glycol (PEG)—create biomimetic surfaces that replicate endogenous protein charge distributions. This stealth effect reduces opsonization, inhibits serum protein aggregation, and extends systemic circulation to promote tumor accumulation [121]. Beyond stabilization, surface functionalization enables active targeting: guanosine conjugation to carboxyl groups on PCL-SS-PEG nanoparticles facilitates choriocarcinoma-specific methotrexate delivery, enhancing tumor uptake while minimizing off-target toxicity [122]. Polymer coatings also impart stimulus-responsive behaviors; for example, *N*-isopropylacrylamide coatings enable thermo-responsive drug release for spatiotemporal control [123]. Collectively, strategic polymer coating addresses critical nanocarrier limitations by prolonging circulation, enhancing tumor specificity, and enabling controlled release. When selecting coating strategies, biocompatibility screening is imperative to prevent biological toxicity. Prioritizing highly biocompatible materials ensures clinical viability while overcoming inherent nanoparticle limitations.

### 4.2. Cell Membrane Coating

**Figure 7:**
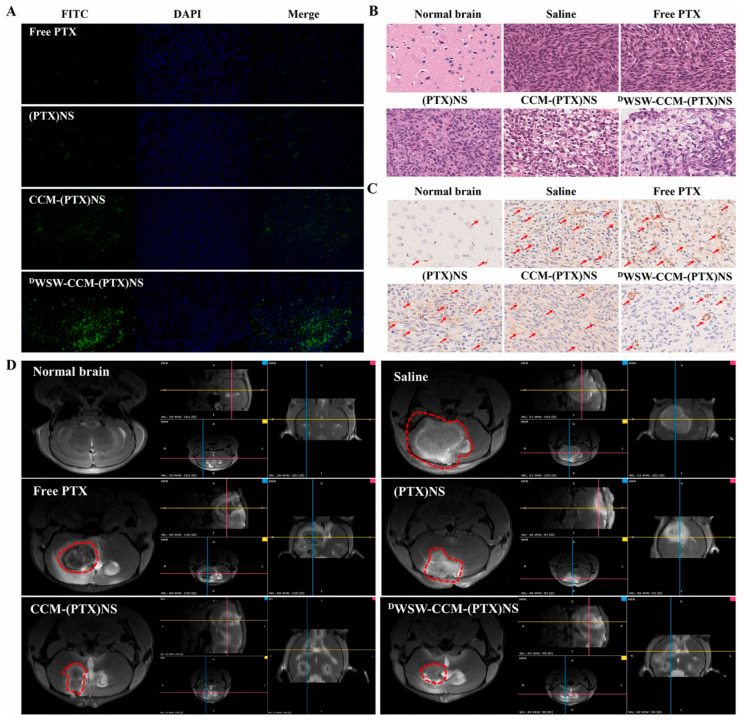
The enhanced anti-glioma efficacy of dual-targeted biomimetic nanosuspensions (“WSW-CCM-(PTX)NS) in C6 tumor-bearing mice. (**A**) TUNEL staining showed that WSW-CCM-(PTX)NS induced the highest degree of tumor cell apoptosis; (**B**) CD31 staining revealed that WSW-CCM-(PTX)NS achieved the lowest tumor microvessel density; (**C**) H&E staining demonstrated that WSW-CCM-(PTX)NS resulted in the smallest tumor volume; (**D**) MRI imaging confirmed that WSW-CCM-(PTX)NS achieved near-complete tumor elimination. Adapted from [126], with permission from KeAi Publishing (2021).

Biomimetic cell membrane-coated nanoparticles represent an emerging frontier in targeted drug delivery, exhibiting high biocompatibility, low immunogenicity, prolonged circulation, and intrinsic homotypic targeting capabilities by mimicking extracellular vesicles [124]. Derived from natural cellular sources, these nanoparticles leverage homologous targeting through cancer cell membrane-expressed adhesion molecules (*N*-cadherin, galectin-3, EpCAM) that facilitate multicellular aggregation [125]. Fan et al. pioneered a dual-targeting strategy by engineering glioma cell membrane-camouflaged paclitaxel nanosuspensions WSW-CCM-(PTX)NS further modified with a brain-penetrating D*D*WSW peptide [126]. As demonstrated in Figure 7, this biomimetic platform achieved superior anti-glioma efficacy in C6 tumor-bearing mice, evidenced by significantly enhanced tumor apoptosis (TUNEL), reduced angiogenesis (CD31), diminished tumor volume (H&E), and minimal residual tumor mass (MRI) compared to unmodified nanosuspensions and free paclitaxel controls. This integrated approach uniquely overcomes two physiological barriers—blood–brain barrier (BBB) penetration via peptide-mediated transcytosis and blood–brain–tumor barrier (BBTB) traversal via homologous targeting—resulting in complete tumor suppression and extended survival. Cui et al.’s conceptual “Trojan horse” nanoparticles complement this work, demonstrating multimodal imaging capabilities [127]. Fan et al.’s innovation lies in synergizing carrier-free high drug loading (≈100%) with active ligand modification to achieve unprecedented glioma-specific delivery efficiency. Despite superior targeting over liposomes, critical limitations impede clinical translation: membrane protein profiles remain poorly characterized, homologous targeting mechanisms require further elucidation, and inherent manufacturing challenges—including low yields, storage instability, and batch-to-batch variability—require urgent resolution.

### 4.3. Platelet Membrane Coating

**Figure 8:**
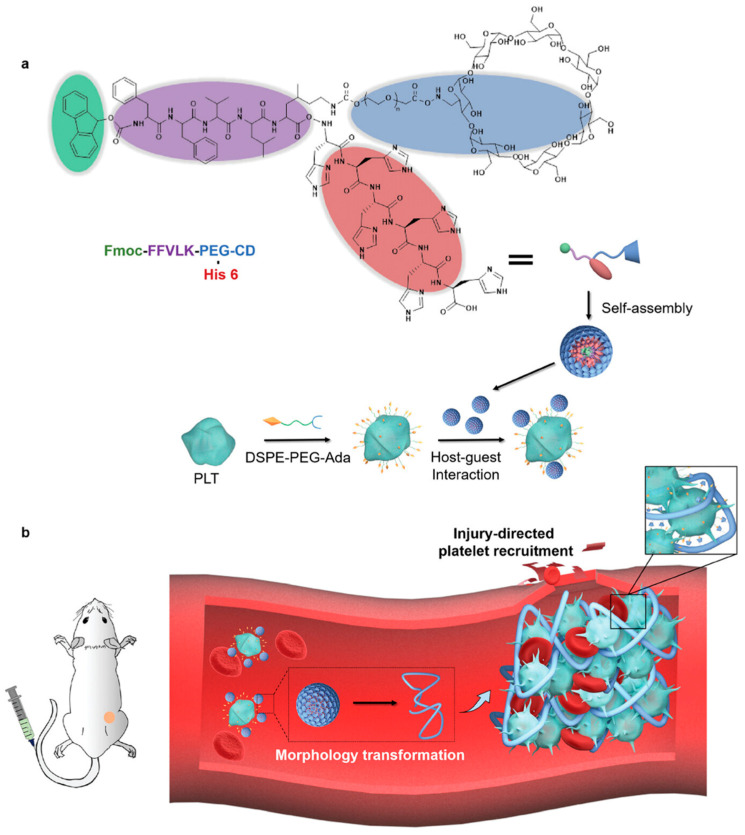
A scheme of supramolecular platelet–nanoparticle conjugates for tumor targeting and in situ coagulation to embolize tumor vessels. (**a**). Supramolecular conjugation enables stable anchoring of morphology-transformable nanoparticles onto platelet surfaces; (**b**) platelet–nanoparticle conjugates target tumor vasculature and undergo acid-triggered fibrin mimetic coagulation. Adapted from [130], with permission from Wiley (2025).

Platelet membrane-coated nanoparticles possess immune evasion capabilities. Through high-affinity interactions between P-selectin on platelet membranes and CD44 receptors on tumor cells, they specifically capture circulating tumor cells (CTCs) that have escaped macrophage uptake in blood and lymphatic circulation, thereby enhancing tumor retention and minimizing immunogenicity [128]. These advantages have driven significant research interest in platelet-based nanocarriers. Wang et al. engineered autologous platelets by loading siRNA-encapsulated nanoparticles into them. These modified platelets homed to renal injury sites, releasing siRNA to achieve target protein knockdown—demonstrating platelet-directed precision delivery [129]. Separately, Li et al. leveraged platelets’ innate vascular targeting and coagulation functions. By integrating self-assembling nanoparticles onto platelet surfaces, they enhanced controlled coagulation/aggregation to deliberately occlude tumor vasculature. This embolotherapy strategy effectively suppressed tumor growth, metastasis, and recurrence (Figure 8) [130]. Zhu et al. developed a self-amplifying tumor-targeting system by conjugating vessel-disrupting agent-loaded nanoparticles with platelets. Exploiting platelets’ tumor-homing properties and coagulation cascades, this platform significantly improved tumor-specific delivery and therapeutic efficacy [131].

### 4.4. Targeted Ligand Coating

Targeted ligand design for nanoparticle surface modification constitutes a core technology in nanomedicine and bioengineering. Its significance lies in the precise regulation of nanoparticle functionality, the enhancement of biomedical application efficacy, and the optimization of safety and controllability [132]. Targeting ligands (e.g., antibodies, peptides, nucleic acid aptamers, glycans, and other biomolecules) are conjugated to nanoparticle surfaces via chemical coupling or physical adsorption. These ligands mediate nanoparticle recognition and binding to specific receptors on target cells or tissues, thereby achieving critical targeting effects [133,134]. While conventional nanoparticles rely on passive targeting through the enhanced permeation and retention (EPR) effect—enabling accumulation in highly permeable pathological tissues such as tumors—they lack specificity for distinct cell types or subpopulations [135]. In contrast, targeting ligands employ a ‘lock-and-key’ binding mechanism (e.g., antibody–antigen interactions) to significantly improve targeting accuracy at cellular and subcellular levels. This approach enables precise discrimination between tumor and normal cells, allows for identification of specific stem cells or immune cell subtypes, and ultimately overcomes the limitations of passive targeting [136].

Felix et al. developed HER3-targeting nanobioparticles capable of penetrating the blood–brain barrier (BBB) to deliver therapeutics to brain tumors. In vitro and in vivo studies demonstrated stable assembly, specific binding to HER3-positive tumor cells, significant accumulation within brain tumors, potent tumor growth inhibition, and low systemic toxicity. Compared to traditional BBB transport systems for tumor therapy, these nanobioparticles exhibited superior therapeutic efficacy and safety, offering a promising clinical strategy for targeted intracranial tumor treatment [137]. Self-assembled nanoparticles composed of 10B-containing copolymers and PD-L1 siRNA were stabilized via disulfide cross-linking while simultaneously conjugating cRGD ligands to their surface for tumor-specific targeting. Subsequently, these nanoparticles dissociated under high concentrations of GSH and ATP, enabling the retention of 10B polymers in tumor cells through intracellular glycoconjugate binding, thereby achieving high tumor enrichment concentrations [138]. Tang et al. designed a multifunctional nanocomposite using ultrafine palladium nanoparticles encapsulated in molecular cages. By conjugating glucose oxidase and hyaluronic acid–nucleic acid aptamers for tumor targeting, the system achieved responsively activated bio-orthogonal catalysis and multi-enzyme mimetic activities within the tumor microenvironment. This significantly enhanced catalytic efficiency and leveraged oxidative stress to potentiate chemotherapy, providing an efficient targeted strategy for precision cancer treatment [139].

Targeted ligand design constitutes a core technology enabling precision medicine applications of nanoparticles. Its advancement relies on the convergence of biochemistry, materials science, and computational biology. Future developments will focus on novel ligand systems exhibiting high specificity, low immunogenicity, and environmental responsiveness. When integrated with intelligent nanocarriers, these systems are expected to enable significant breakthroughs in precision oncology and immunomodulation therapies.

In summary, surface engineering strategies—including polymer coatings, biomimetic membranes, and targeted ligands—critically enhance nanoparticle specificity and functionality. Future advancements necessitate overcoming physiological barriers, developing next-generation multifunctional platforms, and establishing standardized characterization protocols. Integrating intelligent design with dynamic biointerfacial control will be pivotal for realizing precision nanomedicine in oncology and immunomodulation therapies.

## 5. Combination Therapy Strategy Based on Multifunctional Nanoparticles

Nanoparticle carriers offer significant advantages over combinations of free drugs for multidrug delivery. Unlike concurrent administration of free drugs—which exhibit divergent in vivo pharmacological behaviors (e.g., pharmacokinetics, biodistribution, and stability) [140]—nanoplatforms enable co-encapsulation of combinatorial drugs. These systems achieve homogeneous drug action through controlled release mechanisms that synchronize drug delivery and distribution. Crucially, nanoplatforms allow for independent adjustment of release kinetics for each drug [141,142,143], a capability unattainable with free-drug combinations. Furthermore, stimuli-responsive nanocarriers can be engineered for tissue- or cell-specific drug release, enhancing therapeutic efficacy while minimizing off-target toxicity [89,99].

### 5.1. The Co-Delivery of Chemotherapy Agents and Biological Agents

Chemotherapy remains the most widely utilized cancer treatment [144] but faces significant limitations including systemic toxicity and drug resistance [145]. Co-delivery of therapeutic biomolecules—such as targeting ligands, transporter proteins [146], or gene inhibitors [147], with chemotherapeutic agents via nanocarriers can overcome tumor resistance. For example, Dong et al. co-loaded DNA sponges and doxorubicin hydrochloride (DOX) into ZnO nanoparticles. These nanoparticles first targeted ovarian cancer-specific surface markers, then entered tumor cells to cleave DNA via enzymatic activation, thereby enhancing chemosensitivity [148]. Nanostructures address delivery challenges for biological agents like siRNA, which exhibit inherent susceptibility to enzymatic degradation [149]. Diverse nanoparticle platforms now enable stable siRNA delivery [129,150]. Deng et al. encapsulated PD-L1 siRNA in 10B-containing nanoparticles to augment boron neutron capture therapy (BNCT). The siRNA suppressed BNCT-induced PD-L1 upregulation, simultaneously activated T-cell immunity, inhibited DNA repair mechanisms, and generated potent anti-tumor immune responses against distal/metastatic lesions [138].

### 5.2. The Co-Delivery of Chemotherapy Agents and Photosensitizers/Sonosensitizers

Photodynamic therapy (PDT) is a promising cancer treatment modality recognized for its exceptional tumor selectivity and favorable safety profile. It utilizes photosensitizers that, when excited by specific wavelength light, transition from ground state to excited state. Subsequent energy transfer generates cytotoxic reactive oxygen species (ROS), disrupting tumor cell structure and function. For instance, Xv et al. demonstrated enhanced therapeutic efficacy by co-delivering DNA-damaging chemotherapeutics and DNA-repair-inhibiting siRNAs to tumor cells. This combination strategy synergistically increased cancer cell sensitivity to chemotherapy and achieved superior tumor suppression compared to monotherapy [151]. Separately, Dubrova et al. leveraged the photothermal properties of magnetite nanoparticles to achieve near-infrared (NIR) light-controlled hyperthermia. When efficiently accumulated within tumor spheroids, these nanoparticles enabled precise thermal modulation. By optimizing nanoparticle concentration and laser power, temperatures > 44 °C induced significant cell death, while sub-lethal heating (<44 °C) permitted partial tumor cell recovery [152].

Sonodynamic therapy (SDT) is emerging as a promising tumor treatment modality that utilizes ultrasound to activate sensitizers for reactive oxygen species (ROS) generation, inducing tumor cell death. SDT offers significant advantages including no acquired resistance, repeatable administration, and minimal side effects [153]. Sensitizers are broadly classified as organic or inorganic compounds (Table 3). For instance, Huang et al. polymerized and loaded degradable and oxidatively sensitive polymers, sensitizers, and polyphenol-structured polymers with Cu^2+^, which were enriched in the tumor site, and generated ROS under ultrasound, which facilitated the degradation of the nanoparticles and the release of Cu^2+^, triggering copper death (cuproposis) and activating anti-tumor immunity [154]. Moreover, SDT not only offers greater tissue penetration depth but also triggers rapid drug release for synergistic ultrasound chemotherapy. Wu et al. controlled the release of chemotherapeutic drugs through the reactive oxygen species (ROS) response, whereby upon ultrasound stimulation, the intrinsic thioredoxin junctions within the nanoparticles were cleaved by acoustic sensitizer-generated ROS in order to promote the release of paclitaxel and induce apoptosis in tumor cells [155].

### 5.3. Chemotherapy Drugs and Imaging in Combination

Imaging-guided chemotherapy represents a pivotal paradigm in precision oncology, synergizing tumor localization, disease burden quantification, and therapeutic response monitoring with nanocarrier-mediated drug delivery to optimize treatment outcomes [180,181,182]. This approach transcends mere technological integration, marking a transformative shift from empirical treatment to precision medicine [183,184,185]. By leveraging imaging to visualize tumor biology and nanoparticle systems for spatially controlled drug deployment, this strategy not only enhances therapeutic efficacy but also establishes a novel paradigm for managing advanced and treatment-resistant malignancies [183,186,187].

For example, Suman et al. developed DOX-loaded 125I-CPDANP-Dox nanoparticles exhibiting enhanced cytotoxicity in triple-negative breast cancer (TNBC) cells [133]. SPECT imaging and biodistribution studies confirmed rapid blood clearance and significant tumor regression in 4T1 tumor-bearing mice [188]. Meanwhile, Chen et al. engineered Au@DOX/CQ@TPP-DCD nanoparticles that selectively target mitochondria under NIR irradiation, generating ROS and photothermal effects while releasing DOX to directly kill tumor cells and inhibit pro-survival autophagy, collectively achieving 75% tumor regression through enhanced intratumoral accumulation [189]. In parallel, Wang et al. created magnetically driven bionic nanorobots (MDNs) enabling MRI-guided diagnosis, precise tumor targeting, and therapeutic delivery, validated via in vivo imaging and histopathology [190]. Qu et al. designed biodegradable MMGF nanoparticles incorporating O_2_-generating sensitizers to amplify ROS production and potentiate chemotherapy efficacy while reducing systemic toxicity [191]. Multifunctional magnetic Fe_3_O_4_@Au@Bi_2_S_3_ nanoparticles exhibit strong magnetic targeting and enhanced computed tomography (CT) imaging when exposed to an external magnetic field, thereby improving the accuracy of tumor therapy. In vitro and in vivo studies showed that the nanoparticles were effective in inducing apoptosis in cancer cells and inhibiting tumor proliferation, demonstrating excellent anti-tumor properties [192]. In addition, Yang et al. established AuNR@SiO_2_@MnO_2_@DNA nanoparticles to demonstrate a designed multimodal imaging-guided ordered multitherapeutic strategy in lung cancer cells A549, providing an important and promising tool for clinical oncology treatment [193].

The integration of imaging and chemotherapy is evolving beyond mere technological overlay toward bio-intelligent responsiveness, fundamentally reshaping oncology through innovations in multimodal imaging, dynamic nanomaterial modulation, and AI-driven therapeutic decision support [194,195,196,197]. To translate imaging-guided precision chemotherapy from laboratory discovery to clinical practice, future efforts must prioritize material standardization, cost-effective scalability, and interdisciplinary collaboration. This translational focus will enable the delivery of efficient, low-toxicity, patient-tailored treatment regimens that address unmet clinical needs [198,199].

In summary, nanoparticle co-delivery systems enable spatiotemporal orchestration of combination therapies through synchronized pharmacokinetics and stimuli-responsive release. Future advancements require engineering intelligent nanoplatforms that dynamically coordinate multi-tiered therapeutic cascades while integrating real-time imaging feedback. Achieving clinically viable synergistic regimens necessitates overcoming biological barriers and optimizing bio-responsive material design to precisely regulate therapeutic synergy within tumor microenvironments.

## 6. Conclusions

Current nanotechnology research in oncology is advancing substantially beyond single-drug delivery toward developing sophisticated nanoplatforms for co-loading and precision delivery of multiple therapeutics. The core objective of multidrug co-delivery strategies is to exploit synergistic drug interactions—including chemo-sensitization, immune activation, anti-angiogenesis, and multidrug resistance reversal—to enable multi-pathway tumor eradication while suppressing recurrence. Researchers are engineering intelligent nanocarriers with spatiotemporally controlled release capabilities to ensure optimal drug ratios are delivered within specific biological timeframes at target sites. Such precision nanoengineering enhances therapeutic efficiency through improved tumor accumulation, promoted cellular internalization, and minimized off-target effects, while simultaneously reducing systemic toxicity by increasing focal bioavailability and decreasing normal tissue exposure. This dual-advantage paradigm advances oncology toward high-efficacy/low-toxicity standards, representing a transformative shift from monofunctional systems to multifunctional theranostic platforms capable of addressing tumor heterogeneity and complexity.

Despite promising advances, clinical translation faces significant bottlenecks. Scalability and reproducibility challenges persist across nanocarrier classes: albumin-based systems (exemplified by FDA-approved Abraxane^®^) confront limited human serum albumin availability; liposomes exhibit storage instability including shape deformation and osmiophility loss; polymer micelles demonstrate compromised tumor penetration and insufficient clinical efficacy; dendrimers show manageable toxicity in phase II trials but require further safety optimization; inorganic nanoparticles and metal–organic frameworks (MOFs) present dose-dependent toxicity and unpredictable degradation. Patient-specific variables—including tumor microenvironment heterogeneity and immune status—further necessitate personalized design solutions not yet addressed by current platforms.

The coming decade will focus on overcoming these barriers through standardized GMP protocols, AI-optimized nanocarrier design, and biomarker-guided patient stratification. Advancements in continuous-flow nanofabrication will enhance batch-to-batch reproducibility, while modular “plug-and-play” platforms may accommodate diverse payloads. Regulatory frameworks must evolve to address nanomaterial-specific safety assessments, particularly concerning long-term biodistribution. The convergence of nanotechnology with real-time imaging and multi-omics diagnostics will ultimately enable adaptive nano-therapeutics capable of overcoming therapeutic resistance.

This review synthesizes cutting-edge advances in tumor-targeted nanoplatforms, offers critical analyses of multifunctional co-delivery systems, and comparatively evaluates polymeric, lipid-based, biomimetic, inorganic, and MOF-derived carriers, providing strategic insights to guide future research in the field.

## Figures and Tables

**Table 1:** Some designed MOFs and their special functions.

Name	In-Base	Character	Function	Ref.
Fe(TCPP)-MOF	Fe	Amplify ROS generation	Pyroptosis	[101]
ZIF-8	Zn	pH response	Drug release	[102,103]
ZMCH	Zn	Tumor-specific delivery and local drug release	Inhibiting tumor metastasis	[104]
ZIF-67	Co	Catalyze hydrogen peroxide (H_2_O_2_)	Damage cell structure	[105,106]
MIL-88A	Fe	Magnetic resonance imaging (MRI) properties	MRI contrast	[107]
Cu@MIL-101@PMTPC	Fe	Multiple cascading synergistic therapeutic	Precision tumor intervention	[108]
TPP-UCNPs@MOF-Pt	Zr	Catalytically convert intracellular H_2_O_2_ to generate O_2_	Improvement in the hypoxic microenvironment	[109]
bTiO2@TA/Fe	Ti	GSH depletion and chemodynamic therapy	Induce cancer cell ferroptosis	[110]
UCNP@MOF	Cu	Amplified ROS effects and unlocking infiltrating T cells	Tumor eradication	[111]

**Table 2:** The advantages and disadvantages of key nanocarrier systems.

Category	Advantages	Disadvantages	Ref.
Polymer nanoparticles	Sustained release; controlled drug release	Highly cationic nature results in poor biocompatibility	[113]
Liposomes	Good biocompatibility	Difficulties in mass production and storage	[65]
Extracellular vesicles	Good biocompatibility; good targeting	Difficulties in isolation, purification, and mass production; differences among products of the same batch The recognition and function of the surface substances are not clear	[14,69,80]
Inorganic nanoparticles	Outstanding photothermal/magnetic performance; multimodal diagnosis and treatment	Metal ion toxicity caused by off-target behavior	[86]
Metal-organic frameworks	High drug loading; pH-responsive	Metal ion toxicity caused by off-target behavior	[114]

**Table 3:** Types of nano sonosensitizers and their efficacy.

	Category	Nano Sonosensitizer	Efficacy	Ref.
Organic sonosensitizers	Porphyrins	Zr-HMME-PEG-F3	Promotes anti-tumor immunity by suppressing tumor metastasis	[156]
		R@S/SS-NP_H&D_	Provides GSH depletion and amplified ROS generation capabilities	[157]
		HP/CP	Excellent stability, acoustic responsiveness, good tumor targeting and permeability, and efficient sonotoxicity and immune activation	[158]
		CuTA-Ce6	Enhanced tumor cytotoxicity and immunotherapy effect by US	[159]
		MnTTP-HSA	Realizes real-time monitoring of molecular accumulation and tumor targeting for precision theranostic SDT	[160]
	5-Aminolevulinic acid	SPEC5	Achieves robust sensitizer accumulation and enhances SDT efficacy	[161]
	Phthalocyanines	PAMSN	This platform can effectively treat orthotopic liver cancer in a murine model while in vivo monitoring of ROS and detection of cavitation are enabled	[11]
		CuPc-Fe@BSA	Not only has great anticancer effects but also stimulates an anticancer immune response to fight against metastasis and cancer recurrence	[162]
		IrPc NPs	It exhibits good biocompatibility in vitro, can inhibit tumor growth in 4T1 tumor-bearing mice, and enables controllable response by timely ultrasound (US) irradiation during treatment	[163]
	Indocyanines	FA-ICG&MnOx@HSA	Targeting and alleviating tumor hypoxia and improving the tumor immune microenvironment	[164]
		Exo-M (ICG/FX11)	Effectively treats hypoxic tumor cells via combined SDT and energy-depleting chemotherapy	[165]
		IR780-NDs	Mitochondria-targeted and multimodal imaging-guided SDT can be achieved	[166]
	Natural products	BBR NPs	Showing anti-tumor effects both in vitro and in vivo, and its potential mechanisms might be related to inhibiting PI3K-AKT-mTOR signaling pathways and blocking tumor blood vessels	[167]
		APHB NPs	Novel safe and precise NIR FL imaging and SDT agents for deep-seated tumor therapy	[168]
Inorganic Sonosensitizers	Noble metal-based	Janus Au-MnO	Effectively guided synergistic SDT/CDT for deep orthotopic liver tumors	[169]
		(QD@P)R	Utilized the catalase enzyme of the RBC membrane to relieve tumor hypoxia, thereby further enhancing the SDT effect on the tumor under the guidance of fluorescence imaging	[170]
		PtCu_3_	Can pave a new way for imaging-guided in situ TME-responsive CDT-enhanced SDT triggered by US irradiation for deep-seated tumors	[171]
		Pt-MOCs	Effectively produces reactive oxygen species and exhibits superior cytotoxicity for tumor cells	[172]
	Transition metal-based	PEG-TiO1 + x NRs	Because of their efficient passive retention in tumors post intravenous injection, PEG-TiO1 + x NRs can be used as sonosensitizers and CDT agents for highly effective tumor ablation under US treatment	[173]
		TiB_2_@CM-RGD	Effectively crosses the BBB and accumulates in tumor sites, and significantly inhibits tumor growth after US irradiation	[174]
		MnVO_3_	MnVO3 may serve as a highly efficient, low-toxicity, and biodegradable sonosensitizer for cancer SDT	[175]
		D-ZnO-PEG NPs	The simultaneously endowed multiple ferroptosis and synergistically enhanced SDT achieved high in vivo tumor suppression efficiency	[176]
	Carbon-based Si-based	Cu-CDs	Exhibit excellent permeability through the blood–brain barrier and potent anti-tumor activity	[177]
		Si-Pt NCs	The mild photothermal effect of Si-Pt NCs further improves SDT and CDT activity and improves the combined cancer therapy	[178]
		Algae@SiO_2_	The significant suppression of tumor growth in mice bearing a 4T1 tumor successfully demonstrates the promising anti-tumor effect of Algae@SiO_2_-mediated synergistic therapy	[179]

## Data Availability

Not applicable.
